# Use of Multi-Scale Investigation to Evaluate Adhesion Performance of Warm-Mix Polymer-Modified Asphalt

**DOI:** 10.3390/ma16010287

**Published:** 2022-12-28

**Authors:** Ping Li, Ziran Wang, Bo Men, Xiaopeng Ma, Guoqi Tang, Riran Wang

**Affiliations:** 1 Henan Xuxin Expressway Co., Ltd., Zhumadian 463000, China; 2 Yellow River Laboratory, Zhengzhou University, Zhengzhou 450001, China; 3 Guolu Gaoke Engineering Technology Institute Co., Ltd., Beijing 100083, China

**Keywords:** warm mix additives, adhesion property, Macro and micro tests, surface free energy, pull-off test, contact angle

## Abstract

Warm Mix Asphalt (WMA) technology can effectively reduce carbon emissions and energy consumption during road project construction. However, it may have a negative impact on the binding properties of asphalt mixtures. In order to effectively evaluate the adhesion performance of asphalt binders and aggregates under the combined influence of WMA and traditional polymer-modified asphalt, this paper provides a comprehensive evaluation at the micro and macro levels. The adhesion between three different modified asphalts (warm mix crumb rubber/ Styrene-Butadiene-Styrene (SBS) composite modified asphalt, warm mix crumb rubber asphalt, and warm mix SBS modified asphalt) and two different aggregates (limestone and granite) under both virgin and short-term aging conditions were analyzed. Regardless of the type of modified asphalt, the results showed that limestone aggregates have better adhesion properties with asphalt binders. In addition, the short-term thermal oxidation aging behavior is conducive to enhancing the asphalt-aggregate adhesion characteristics. Furthermore, WMA additives, crumb rubber, and SBS compound modification can improve the adhesion performance between asphalt and aggregate.

## 1. Introduction

Moisture damage is one of the most common pavement ailments and can accelerate the occurrence of asphalt road damage [1,2,3]. Therefore, to ensure the service function of asphalt roads and improve the bonding properties and moisture susceptibilities of asphalt-aggregate systems, modification methods with strong adhesion-promoting effects have been applied on a large scale [4,5,6,7]. Polymer-modified asphalt is widely used in asphalt pavement construction all over the world because of its good durability. However, there are numerous issues with its construction, including incompatibility, high energy use, carbon emissions, and environmental degradation. Thankfully, these problems can be resolved with the development of WMA.

WMA produces asphalt at temperatures 20–40° lower in comparison to Hot Mix Asphalt (HMA) [8]. In addition, a significant reduction in pollutant and greenhouse effect gas emissions has been reported [9]. Yue et al. [10], Yu et al. [11], and A. M. et al. [12] found that the addition of warm mix materials to polymer-modified asphalt can successfully lower the required temperature and carbon dioxide emissions during the construction process, ultimately reducing environmental pollution and saving energy. However, the moisture damage of WMA mixtures might be more complicated than HMA mixtures [13]. It is considered that some factors within WMA mixtures might influence moisture susceptibility, such as aggregate type, aggregate moisture content, asphalt grade, aging, modifying agent, etc.

At present, many scholars often choose some macro-scale physical-mechanical methods when evaluating the properties of asphalt mixtures. Wang et al. [14] used different macroscopic tests to study the antiaging performance of graphene oxidation or carbon nanotube-modified asphalt binders. Zhang et al. [15] carried out the frequency sweep test, fatigue-healing test, and complete process monitoring test of self-healing behavior to assess the impact of rock asphalt on the self-healing characteristic of asphalt mastic. Wang et al. [16] focused on comparing the performance of steel slag wastes and natural limestone in terms of micro-mechanism, macro-fatigue behavior, and microwave heating healing capability. Liu et al. [17] performed macroscopic tests to determine the optimum content of the SBR modifier agent and evaluated the comprehensive road performance of SBR-BRA-modified asphalt mixtures. Although the macroscopic method is simple and convenient to operate, it is highly subjective, poorly based on theory, and has poor field repeatability. Therefore, it is necessary to find a theoretical basis close to the field conditions of the evaluation method to accurately guide the engineering practice. As asphalt properties can be analyzed by computers, H. H. et al. [18] used Multi-Expression Programming (MEP) to develop empirical predictive models for the Marshall parameters. Many other studies have provided similar ideas..

In recent years, theoretical models of surface free energy based on micromechanical techniques have been used to assess the bonding qualities of composites made of asphalt. The surface free energy (SFE) method is an effective way to evaluate and estimate the moisture susceptibility potential of an asphalt mixture [19,20] and could directly address SFE components: work of adhesion and deboning of bitumen and aggregate [21,22,23,24]. Cheng [25] et al. analyzed the relationship between the surface energy index and macroscopic components and pointed out that the surface energy approach can be utilized to assess the water sensitivity of asphalt mixes. In order to more precisely examine the adhesion characteristics of asphalt and aggregate, Bhasin [26] measured surface energy. He discovered that the lithology of the aggregate has a significant role in the adhesion characteristics of the asphalt-aggregate system. According to Wang [27] et al., the adhesion performance between asphalt and aggregate may be assessed using the adhesion work estimated using surface energy theory. When there is moisture action, the damage to the adhesion type between asphalt and aggregate predominates. Xiao [28] et al. conducted freeze-thaw splitting tests on asphalt mixtures and found that their freeze-thaw splitting strength ratio TSR values correlated well with the water stability evaluation index ER proposed by Bhasin [25].

**Figure 1:**
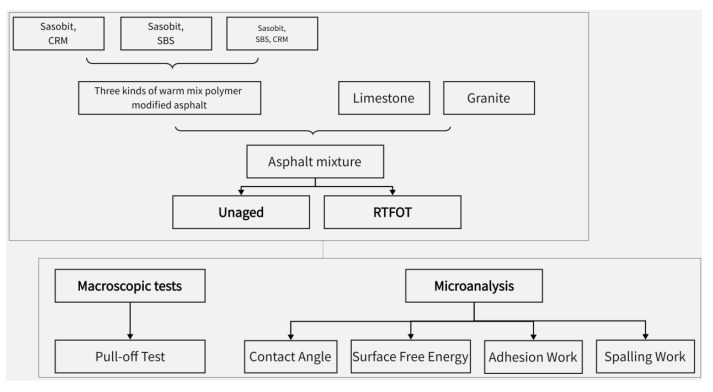
Flowchart of the study.

Few current studies have addressed whether the adhesion properties between the asphalt binder and aggregate under the combined action of warm mixes and polymer modifiers meet the water stability requirements. This paper attempts to investigate the effects of three factors, namely, aggregate type, modifier, and aging, on the asphalt-aggregate adhesion properties. Based on this, the mastic powder, SBS modifier, and wax-based organic warm mixer Sasbiot were selected to prepare a warm mix polymer-modified asphalt binder. Limestone and granite were selected as typical aggregates. Based on micro-mechanical methods and macro-mechanical tests, the adhesion performance of warm-mix polymer modified asphalt-aggregate was studied. At the same time, the effect of thermal-oxidative aging on the adhesion performance of warm-mix polymer modified asphalt-aggregate was evaluated. Figure 1 shows the flow chart of this study.

## 2. Materials and Methods

### 2.1. Materials

To begin, 70# matrix asphalt, SBS modifier, crumb rubber modifier (CRM), and WMA additives Sasobit were chosen for this study as raw materials to prepare warm mix polymer-modified asphalt. Limestone and granite were selected as the representative aggregates of different lithologies.

#### 2.1.1. Base Asphalt

According to the “Highway Engineering Asphalt and Asphalt Mixture Test Procedure” (JTG E20-2011), the main technical indicators of the matrix asphalt were tested, and the test results are shown in Table 1.

#### 2.1.2. WMA Additives

The most widely used classification differentiates warm mixes by the technology used and divides them into three categories: (i) foaming processes; (ii) addition of organic additives; and (iii) addition of chemical additives.

Sasobit is an organic warm mix additive developed by Sasol-Wax in South Africa, which has been widely used around the world. Sasobit promotes the elastic properties and deformation resistance of the binder, giving it better resistance to water damage, fatigue failure, and rut [29,30]. The main performance index of Sasobit is given in Table 2.

#### 2.1.3. Polymer

SBS, as a favorable modifier, has been verified and widely utilized in the construction of asphalt pavement. Some studies validated that the addition of SBS modifiers with asphalt binder could remarkably increase the viscosity of the asphalt binder [7]. CRM has reported many advantages; for instance, improved bitumen resistance to rutting due to high viscosity, a high softening point and better resilience, reduction in road pavement maintenance cost, lower fatigue/reflection cracking, and less temperature susceptibility [31,32].

The main technical specifications of the polymer modifiers used in this paper are shown in Table 3 and Table 4.

### 2.2. Preparation of Modified Asphalt

A warm mix of polymer-modified asphalt was prepared by the melt method. According to other studies [11,28,33,34], the specific steps were as follows: First, matrix asphalt was put into the oven at a temperature of 125 ± 5 °C and heated to the flow state. Second, a certain quantity of polymer modifier (SBS, CRM, or SBS + CRM) was added separately, at a temperature of 170 °C, and sheared for 30 min at f 5000 r/min to produce the polymer-modified asphalt. After that, the polymer-modified asphalt was put into the oven at 140 °C for 30 min to stay warm. Finally, the polymer-modified asphalt was taken out, and a certain mass of Sasobit was added and sheared again at 5000 r/min for 30 min to produce the required warm mix polymer-modified asphalt specimens. Table 5 shows the content of each type of additive. Penetration, softening point, and ductility tests were performed on the above-modified asphalts and the results are listed in Table 6.

### 2.3. Short-term Aging Tests

In this research, based on the “Test Procedure for Asphalt and Asphalt Mixture for Highway Engineering (JTG E20-2011)”, the Rolling Thin Film Oven Test (RTFOT) was used to simulate short-term aging. The test procedure was as follows. First, the flowing original polymer-modified asphalt was loaded into special short-term aging bottles. The mass of each bottle was 35 ± 0.5 g. After that, the bottles were put into a rotating thin film oven for 85 min of thermal oxygen aging to simulate the short-term aging of asphalt. During the aging process, the parameters of the rotating film oven were controlled as follows: temperature 163 ± 0.5 °C, airflow 4000 mL/min ± 200 mL/min, and turntable speed 15 ± 0.2 r/min.

### 2.4. Surface Free Energy Methods

The surface free energy is a micromechanical measurement of the material surface that accounts for the energy required to form a new unit area on the surface of materials. There are numerous theoretical models for computing surface free energy. However, according to van Oss [35] and others, the surface energy of objects is composed of a polar component and a dispersive component (non-polar component). The polar component is divided into the Lewis acidic component and the Lewis alkaline component as shown in Equation (1).

(1) $$\gamma =\gamma^{d}+\gamma^{p}=\gamma^{d}+2\sqrt{\gamma^{+}\gamma^{-}}$$

where γ is the surface energy; $\gamma^{d}$ is the dispersive component of the surface energy; $\gamma^{p}$ is the polar component of the surface energy; $\gamma^{+}$ is the Lewis acid component; and $\gamma^{-}$ is the Lewis alkaline component.

When the contact angle of the test liquid and asphalt is known, it can be combined with the surface energy of the test liquid and its components and the surface energy parameters of asphalt can be calculated, as shown in Equation (2).

(2) $$\left(1+\cos\theta \right)\gamma_{l}=2\left(\sqrt{\gamma_{B}^{d}\gamma_{l}^{d}}+\sqrt{\gamma_{B}^{+}\gamma_{l}^{-}}+\sqrt{\gamma_{B}^{-}\gamma_{l}^{+}}\right)$$

where $\gamma_{l}$ is the surface energy of the test liquid; $\gamma_{l}^{d}$ is the non-polar component of the test liquid; $\gamma_{l}^{-}$ is the Lewis alkaline component of the test liquid; and $\gamma_{l}^{+}$ is the Lewis acidic component of the test liquid.

Adhesion work [36] is the energy needed during the water damage process to split the contact surface of asphalt and aggregate into two interfaces. It is based on the surface energy parameters of asphalt and aggregate, as given in Equation (3). Cohesion work reflects the energy needed to establish two interfaces within the asphalt [19], which is equal to twice the asphalt’s surface energy, as indicated in Equation (4).

(3) $$W_{\mathrm{AB}}=\left(1+\cos\theta \right)\gamma_{B}=2\left(\sqrt{\gamma_{B}^{d}\gamma_{A}^{d}}+\sqrt{\gamma_{B}^{+}\gamma_{A}^{-}}+\sqrt{\gamma_{B}^{-}\gamma_{A}^{+}}\right)$$

(4) $$W_{\mathrm{BB}}=2\gamma_{B}$$

where $\gamma_{A}^{d},\gamma_{A}^{+},\gamma_{A}^{-}$ are the dispersion component, Lewis acidic component, and Lewis alkaline component of aggregate surface energy; $\gamma_{B}^{d},\gamma_{B}^{+},\gamma_{B}^{-}$ are the dispersion component, Lewis acidic component, and Lewis alkaline component of asphalt surface energy; $W_{\mathrm{AB}}$ is the adhesion work between asphalt and aggregate; $W_{\mathrm{BB}}$ is the adhesion work inside asphalt; θ is the contact angle; and $\gamma_{B}$ is the surface energy of asphalt.

During the lifespan of the road, water intrusion into the asphalt pavement is inevitable. The adhesion work primarily reflects the adhesion performance of the asphalt-aggregate system under water-free conditions. Water will replace a portion of the asphalt at the asphalt-aggregate interface when there is water intrusion, causing the asphalt and aggregate to be stripped. The peeling power index is employed in this procedure to assess how well the asphalt-aggregate system resists peeling. The higher the number, the worse the resistance. The computation of peeling power is indicated in Equation (5).

(5) $$\begin{array}{cc} W_{\mathrm{ABW}}=-(\gamma_{\mathrm{AW}} & +\gamma_{\mathrm{BW}}-\gamma_{\mathrm{AB}})\\ & =2\sqrt{\gamma_{W}^{+}}\left(\sqrt{\gamma_{B}^{-}}+\sqrt{\gamma_{A}^{-}}\right)+2\sqrt{\gamma_{W}^{-}}\left(\sqrt{\gamma_{B}^{+}}+\sqrt{\gamma_{A}^{+}}\right)+2\sqrt{\gamma_{W}^{d}\gamma_{A}^{d}}+2\sqrt{\gamma_{B}^{d}\gamma_{W}^{d}}-2\gamma_{W}^{d}-2\sqrt{\gamma_{B}^{d}\gamma_{A}^{d}}\\ & -2\sqrt{\gamma_{B}^{+}\gamma_{A}^{-}}-2\sqrt{\gamma_{B}^{-}\gamma_{A}^{+}}-4\sqrt{\gamma_{W}^{+}\gamma_{W}^{-}} \end{array}$$

where $W_{\mathrm{ABW}}$ is the peeling work between asphalt, water, and aggregate; $\gamma_{\mathrm{AW}}$ is the surface energy of aggregate-water interface; $\gamma_{\mathrm{BW}}$ is the surface energy of the asphalt-water interface; $\gamma_{\mathrm{AB}}$ is the surface energy of aggregate-asphalt interface; $\gamma_{W}^{d}$ is the non-polar component of water; $\gamma_{W}^{-}$ is the Lewis alkaline component of water; and $\gamma_{W}^{+}$ is the Lewis acidic component of water.

The adhesion work and spalling work only reflect the adhesion performance of asphalt and aggregates in the absence or presence of water from one side. Bhasin et al. [26] proposed the Energy Ratio (ER) index based on the adhesion work and peeling work, as indicated in Equation (6), to suit the practical objectives of engineering and to evaluate the water stability of the asphalt-aggregate system. The greater the value of ER, the better the adhesion performance between the asphalt and aggregate during the wetting and adaption process.

(6) $$\mathrm{ER}=\left|\frac{W_{\mathrm{AB}}}{W_{\mathrm{ABW}}}\right|$$

Distilled water, glycerol, and formamide were selected as the standard test liquids, and their surface energies and parameters are shown in Table 7.

### 2.5. Pull-off Test

**Figure 2:**
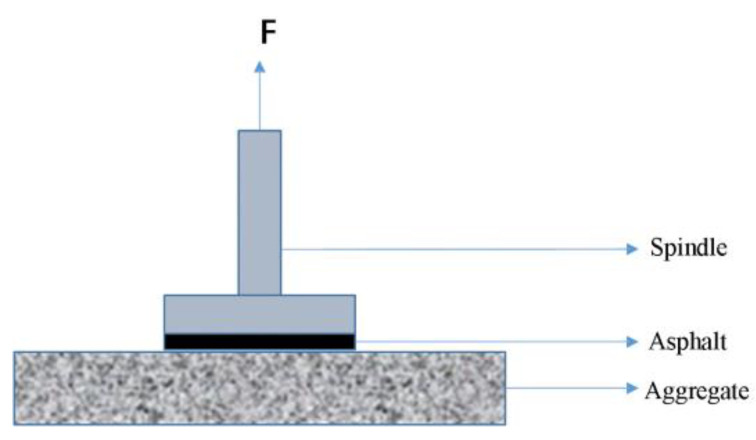
Schematic diagram of the pull-off test [35].

The LGZ-1 pull-off tool, whose operational concept is depicted in Figure 2, was used to conduct the pull-off test.

## 3. Results and Analysis

### 3.1. Micromechanics Surface Free Energy Research

#### 3.1.1. Contact Angle

The laying-drop method was used to measure the contact angles between limestone, granite, and the three test liquids. The results are displayed in Table 8. According to Table 8, limestone mixed with the three test liquids has smaller contact angles than granite, which indicates that the liquid can easily wet the limestone aggregate.

**Figure 3:**
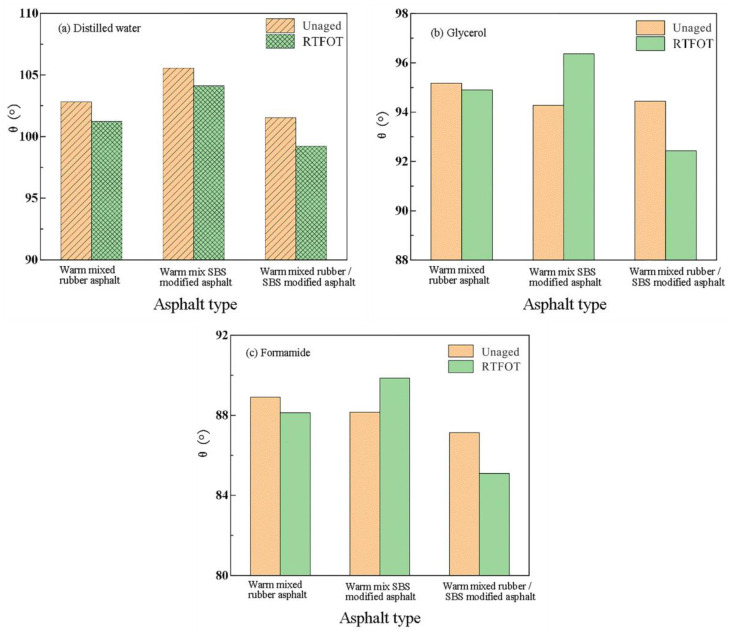
The contact angle of asphalt and polar liquid: (**a**) Distilled water, (**b**) Glycerol, and (**c**) Formamide.

The contact angles of asphalt with the test liquids are shown in Figure 3. It was found that the effect of aging on the contact angle of asphalt specimens with the three test liquids was different. Since the primary goal of this work is to investigate the moisture damage resistance of aggregate and asphalt, the contact angle between the specimens of asphalt with distilled water as the test liquid was selected for further testing. According to Figure 3, the warm mix of polymer-modified asphalt is a typical hydrophobic medium since the contact angle between the material and distilled water is more than 90°. Warm-mix crumb rubber asphalt, warm-mix SBS modified asphalt, or warm-mix crumb rubber/ SBS composite modified asphalt had a smaller contact angle with distilled water after RTFOT aging. This indicates that RTFOT aging decreases asphalt specimens’ hydrophobicity and improves the infiltration of distilled water into the asphalt.

#### 3.1.2. Surface Free Energy

**Figure 4:**
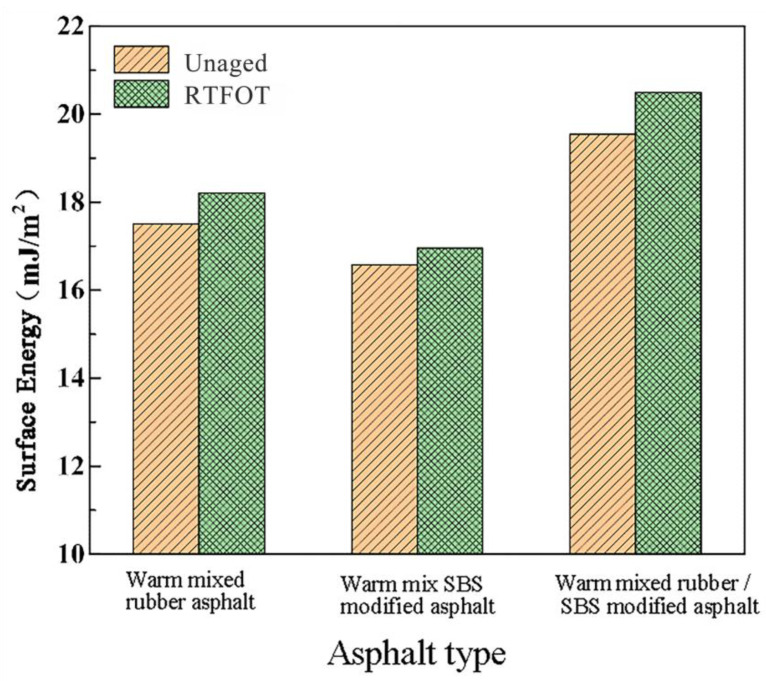
The surface energy of the modified asphalt sample before and after short-term aging.

The surface energy of asphalt and aggregate can be calculated from the contact angle between the liquid and asphalt, the contact angle between the liquid and aggregate, and the surface energy of the three liquids by combining Equation (2). The surface energy of the aggregates and their components are shown in Table 9, and the surface energy of asphalt before and after aging is shown in Figure 4.

From Table 9, we see that the surface energy of limestone and granite are 46.81 mJ/m^2^ and 34.97 mJ/m^2^. Granite is an acidic aggregate, while limestone is an alkaline aggregate. Asphalt contains more acidic constituents per volume than alkaline constituents. As a result, alkaline aggregates cling to asphalt more effectively [37,38]. In addition, the surface energy of limestone is larger than that of granite, which is consistent with the previous findings reported in the literature.

As shown in Figure 4, warm mix crumb rubber/SBS composite modified asphalt ranks above warm mix crumb rubber asphalt and ahead of warm mix SBS modified asphalt in terms of surface energy under the same aging situation. This is related to the modification mechanism of warm-mix polymer-modified asphalt. The mastic powder swells and creates an interfacial transition layer with the asphalt during the polymer modification process, improving the asphalt’s overall performance. Additionally, the fine mastic powder particles in asphalt can spread out internal tensions and increase asphalt’s resistance to breaking. SBS modifiers can physically bond with asphalt during the swelling process so that there is an embedded force between them, improving the cohesiveness of asphalt. The warm-mix crumb rubber/SBS composite modified asphalt demonstrates good cohesive qualities as a result of the interaction between the CRM and SBS modifier, as well as the production of a network structure inside the asphalt. When compared to SBS modification, the mastic powder modification absorbs a lot more light components during the swelling process, increasing the viscosity of the asphalt and improving the cohesiveness effect [39,40].

Since the cohesive power and cracking resistance of asphalt increase with surface energy, it can be said that RTFOT aging aids in enhancing warm mix polymer-modified asphalt’s cracking resistance.

#### 3.1.3. Adhesion Work

**Figure 5:**
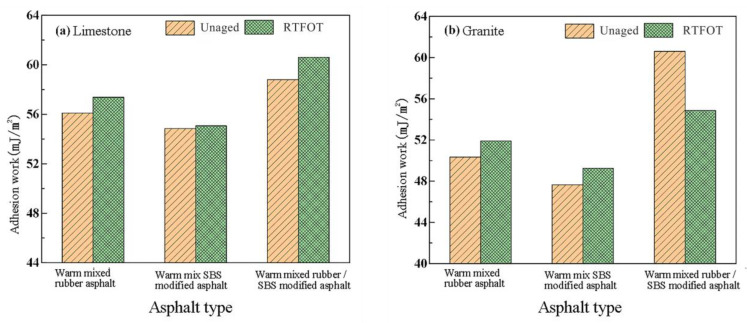
Adhesion work of asphalt and different aggregates before and after aging: (**a**) Limestone and (**b**) Granite.

The adhesion work characterizes the asphalt-aggregate adhesion performance. Figure 5 displays the adhesion work of an asphalt-aggregate before and after aging based on the asphalt and aggregate’s surface energy characteristics.

From Figure 5, it can be seen that the change in adhesion power of the three modified asphalts is basically the same for the same aggregate. After short-term aging, the adhesion power of both types of aggregates and asphalt increased. This suggests that short-term aging contributes to improving the asphalt-aggregate interface’s ability to connect in an anhydrous state and increases the system’s stability. Warm-mix crumb rubber/SBS composite modified asphalt does have a higher adhesive power than the other two varieties of asphalt, whether they contain limestone or granite aggregates, under identical aging conditions. This shows that, compared to a single polymer modification, the combination of a binder powder and SBS modifier can increase the adhesion capacity of warm mix polymer-modified asphalt under aging.

#### 3.1.4. Spalling Work

The asphalt-aggregate peeling work before and after aging was calculated from the surface energy parameters of asphalt and aggregate.

**Figure 6:**
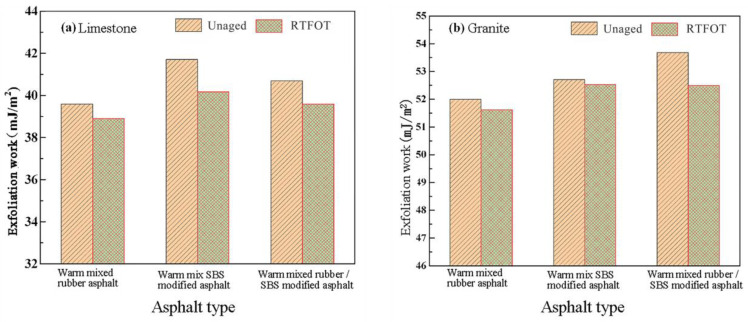
Peeling work of asphalt and different aggregates before and after aging: (**a**) Limestone and (**b**) Granite.

Figure 6 shows that the peeling work of modified asphalt and aggregate gradually decreases with the occurrence of short-term aging behavior. The smaller the peeling work, the better the asphalt-aggregate adhesion performance and the stronger the resistance to peeling in the presence of water. Therefore, it can be concluded that the short-term aging behavior has a positive effect on the stability of asphalt and aggregate in the presence of water.

#### 3.1.5. Energy Ratio

The energy ratio can be obtained by substituting the adhesion and peeling data into Equation (6). Table 10 shows the energy ratio of warm mix polymer-modified asphalt to aggregate before and after aging.

Table 10 shows that the energy ratio tends to rise with short-term aging. When limestone particles are chosen as coarse aggregates, since their energy ratio is always larger than that of granite aggregates, the water stability of asphalt mixes is optimum.

### 3.2. Macro Mechanics-Pull-Off Test Research

The results of the pull-off force between the modified asphalt specimens and the aggregates are shown in Table 11. It can be seen that the pull-off force between the three asphalt binders and the aggregate rises greatly after short-term aging. This is due to the fact that the asphalt’s active components (asphaltene and asphaltene anhydride), which are mostly concentrated in the gum and asphaltene, are related to the stability of the asphalt-aggregate interface. New functional groups, including sulfoxide groups and carbonyl groups, are created inside the asphalt material during the short-term aging process by chemical reactions, increasing the concentration of active substances. As a result, the asphalt and the aggregate are more wettable, which enhances the adhesion at the interfaces [27].

The warm mix crumb rubber/SBS composite modified asphalt had the maximum tensile strength and showed improved bonding ability with aggregates, following the analysis of the change of tensile strength of different types of modified asphalt before and after aging. The pull-off force of the interface between asphalt and limestone is greater than that of granite aggregates when the modified asphalt types are the same. This suggests that the aggregate’s lithology has a significant impact on the interface’s ability to bond.

## 4. Conclusions and Recommendations

Based on the micro mechanical method and macro mechanical characterization method, the following conclusions were obtained by calculating and analyzing the parameters of surface energy, adhesion work, spalling work, and energy ratio of the warm-mix polymer modified asphalt and aggregate system.

(1) The contact angle between asphalt and distilled water decreases with short-term aging, showing that the hydrophobicity of warm-mix polymer-modified asphalt decreases. As a result, the asphalt’s ability to resist water damage is reduced.
(2) When the degree of aging is the same, microscopic tests showed that the sequence of cohesion work is warm mix crumb rubber/SBS composite modified asphalt > warm mix crumb rubber asphalt > warm mix SBS modified asphalt. This suggests that asphalt has better bonding properties. Despite the warm mix rubber/SBS composite modified asphalt showing the highest cohesive power, there is still a need for a thorough cost-benefit analysis in actual engineering applications.
(3) Aging has a considerable impact on asphalt binders. Short-term aging can enhance the adhesion performance of asphalt and aggregate, according to the analysis of adhesion work, spalling work, and the energy ratio between asphalt and aggregate with aging behavior. This improving effect may be related to the further swelling of the modifier in the asphalt during short-term aging. It also might be a result of the aged asphalt’s higher polarity connecting more strongly with the polar material on the stone surface.
(4) When the asphalt type and aging state were the same, analysis of the pull-off test results revealed that the interfacial adhesion between asphalt and limestone aggregates was superior to that of granite. Warm-mix crumb rubber/SBS composite modified asphalt has the best adhesion when the aggregate type and asphalt’s aging state are the same. The conclusions drawn from the microscopic method are congruent with this.

The conclusions presented in this paper are for one selected WMA additive. It is recommended that performance tests of asphalt mixed with different WMA additives be conducted to better understand their behavior. In addition, the cost-benefit research of warm-mix composite modified asphalt must also be carried out.

## Figures and Tables

**Table 1:** Basic technical indexes of base asphalt.

Technical Indicators	Unit	Measured Value	Technical Requirement
Penetration (25 ℃, 100 g, 5 s)	0.1 mm	67.3	60~80
Ductility (5 cm/min, 25 ℃)	cm	>100	>100
Softening point (Ring-and-ball method)	℃	48.3	≥46
Brinell rotational viscosity (135 ℃)	Pa·s	0.45	—

**Table 2:** Main technical parameters of warm mix additives.

Type of Warm Mixing Agent	Technical Indicators	Unit	Measured Value
Sasobit	Flash point	℃	290
	Melting point	℃	100
	Viscosity (135 °C)	Pa·s	5.47 × 10^−3^
	Viscosity (150 °C)	Pa·s	3.26 × 10^−3^
	Penetration (25 °C)	0.1 mm	1
	Penetration (50 °C)	0.1 mm	8
	pH value	—	Neutral
	Solubility (20 ℃)	—	Insoluble

**Table 3:** Basic technical parameters of SBS modifier.

Technical Indicators	Unit	Measured Value
Oil content	%	0.7
Volatiles	%	≤0.7
Tensile strength	MPa	≥18.0
Molecular structure	–	Linear

**Table 4:** Basic technical indexes of CRM.

Technical Indicators	Unit	Measured Value
Particle size	Mesh	60
Density	g/cm^3^	1.13
Carbon black	%	32.76
Moisture content	%	0.61

**Table 5:** Different asphalt modifier addition.

Modified Asphalt	Additive Content (*wt%*)		
	SBS	CRM	Sasobit
4%SBS + 3%Sasobit	4	0	3
15%CRM + 3%Sasobit	0	15	3
3%SBS + 10%CRM + 3%Sasobit	3	10	3

**Table 6:** Modified asphalt conventional technical specifications.

Technical Specifications	Unit	Modified Asphalt		
		4%SBS +3%Sasobit	15%CRM +3%Sasobit	3%SBS +10%CRM +3%Sasobit
Penetration(25 ℃)	0.1 mm	39.9	25.7	34.6
Ductility (15 ℃)	mm	566.0	108.0	170.0
Softening point	℃	85.3	89.8	90.9

**Table 7:** Surface energy and parameters of test solution (mJ/m^2^).

Type of Test Fluid	γ	$\gamma^{d}$	$\gamma^{p}$	$\gamma^{+}$	$\gamma^{-}$
Distilled water	72.80	21.80	51.00	25.50	25.50
Glycerol	64.00	34.00	30.00	3.92	57.40
Formamide	58.00	39.00	19.00	2.28	39.60

**Table 8:** Measurement results of the contact angle between aggregate and test liquid.

Aggregate Limestone	Contact Angle (°)		
	Distilled Water	Glycerol	Formamide
Limestone	65.42	46.57	32.71
Granite	78.54	57.19	48.66

**Table 9:** Surface free energy of aggregate (mJ/m^2^).

Aggregate	γ	$\gamma^{d}$	$\gamma^{p}$	$\gamma^{+}$	$\gamma^{-}$
Limestone	46.81	37.04	9.77	2.13	11.2
Granite	34.97	27.96	7.01	3.92	3.14

**Table 10:** Energy ratio of asphalt and aggregate before and after aging.

Asphalt Type	Unaged		RTFOT	
	Limestone	Granite	Limestone	Granite
Warm mix crumb rubber asphalt	1.417	0.968	1.475	1.006
Warm mix SBS modified asphalt	1.315	0.904	1.371	0.938
Warm mix crumb rubber / SBS composite modified asphalt	1.445	0.990	1.531	1.045

**Table 11:** The pull-off force of asphalt and aggregate before and after short-term aging (MPa).

Asphalt type	Unaged		RTFOT	
	Limestone	Granite	Limestone	Granite
Warm mix crumb rubber asphalt	0.19	0.14	0.31	0.26
Warm mix SBS modified asphalt	0.22	0.17	0.34	0.30
Warm mix crumb rubber / SBS composite modified asphalt	0.24	0.20	0.38	0.33

## Data Availability

Not applicable.
